# Self-Care Recommendations of Middle-Aged and Older Adults with Sickle Cell Disease

**DOI:** 10.1155/2011/270594

**Published:** 2011-08-15

**Authors:** Coretta M. Jenerette, Cheryl Brewer, Ashley N. Leak

**Affiliations:** School of Nursing, The University of North Carolina at Chapel Hill, Campus Box 7460, Chapel Hill, NC 27599-7460, USA

## Abstract

Self-care management is an important part of living with a chronic illness. Sickle cell disease (SCD) is a chronic disease with acute, painful exacerbations that often results in a shortened life expectancy. Some middle-aged and older adults with SCD lived with the disease prior to having a diagnosis and without modern advances. The purpose of this study is to share the self-care recommendations of middle-aged and older adults with SCD. Using descriptive qualitative methods, data were gathered through semistructured interviews from 11 individuals living with SCD, including 6 women and 5 men. Self-care recommendations themes included physiological, psychological, and provider-related. The self-care recommendations may be seen as an additional resource or “words of wisdom” for younger adults with SCD who can use the recommendations to better manage their own disease. Additionally, providers may be able to use these recommendations to inform their practice.

## 1. Introduction

Older African Americans have traditionally been highly respected within their families primarily due to experiences, knowledge, and recall related to historical events that have influenced their lives [1, 2]. Information shared by older African Americans often includes aspects of African American culture such as conditions of slavery, race relations, living standards, challenging economic times, and poor medical conditions. African American elders have also historically responded to health, healthcare, and illness in several ways. Reactions among this population include expressions of perceptions of illnesses and conditions, beliefs related to the origin or cause of illness conditions, reluctance towards the use of prescription medications, use of home remedies, and the importance of spirituality in health, illness, and healing [2, 3]. Moreover, many older African Americans do not adequately and consistently utilize the health care system as a result of past and present discriminatory experiences, stigma, and lack of trust in medical doctors and health care professionals [3–5]. 

In as much as African American elders may possess a wealth of knowledge and information related to historical experiences, it may be useful for health care professionals to engage in reminiscence when interacting with older African Americans [5, 6]. This practice will assist in showing respect toward this population and gaining a better understanding of their cultural views, experiences in life, and health care practices. Moreover, information from elders may be of benefit to younger individuals who may be living with the same illness such as in the case of sickle cell disease (SCD). A recent study conducted by Sanders et al. concluded that the age of adults with SCD was a significant determinant for health care utilization and pain coping patterns of individuals with sickle cell disease [7].

SCD is a chronic condition found primarily in populations of African American and sub-Saharan African descents [8]. The lives of persons with SCD are characterized by frequent and unpredictable pain episodes (resulting from lack of oxygen owing to the occlusion of blood vessels by sickle-shaped red blood cells), numerous hospitalizations for pain or other complications of SCD, and a host of economic and familial burdens [9].

The life expectancy for persons with SCD has increased from 14 years in 1973 to the mid-to-late 40s in 2004, transforming SCD from mainly a childhood disease to a long-term chronic illness [10, 11]. In individuals with SCD, life expectancy is influenced by the type of SCD. In the United States, 65% of the SCD cases are the most severe–homozygous HbSS disease or SCD-SS—while 25% of the cases are heterozygous or SCD-SC with the remaining 10% being comprised of other genotypes such as SCD-S*β* thalassemia [10, 11]. In the Cooperative Study of Sickle Cell Disease focused on the natural history of 3,578 individuals with SCD age newborn to 66 years, Platt et al. found that the average pain rate in SCD-SS was twice that of individuals with SCD-SC [12]. Platt and colleagues reported that the median age of death was 42 years for males with SCD-SS and 48 years for females with SCD-SS while in individuals with SCD-SC, the median ages of death were 60 and 68 years for males and females, respectively [13]. Although this increase in longevity is partly due to advances in the diagnosis and treatment of SCD, people with SCD still often receive inadequate health care. It is not uncommon for patients with SCD to be labeled by healthcare providers as malingerers or manipulators or even drug seekers [14–16].

In the health care practices of individuals living with chronic diseases, self-care activities are very important. For vulnerable, predominantly minority populations, such as individuals with SCD, self-care activities are even more important. In the case of SCD, medical interventions result in substantial costs financially and psychologically. Health-related stigmatization may hinder care-seeking for the acute pain exacerbations of pain—the hallmark of the disease [17]. When individuals with SCD seek care, they often report poor interpersonal healthcare experiences, including poor communication with providers and poorer patient ratings of provider communication are associated with lower trust toward the medical profession among adults with SCD [18]. The majority (80%) of adults with SCD manage episodes of acute pain at home thus often avoiding the health care system [19].

For these reasons, self-care activities are critical to decrease health care costs as well as improve the health status and quality of life for persons living with SCD. By learning how middle-age and older adults with SCD cope with the many challenges of SCD, providers may be better able to address these challenges while also incorporating appropriate self-care strategies into the plan of care of others with SCD. Once SCD has been diagnosed, individuals need to learn to manage symptoms and maintain control over the course of the disease to maintain an acceptable quality of life [20]. The purpose of this paper is to describe self-care recommendations offered by middle-aged and older adults with SCD.

## 2. Methods

The data presented were collected as part of a study wherein we used what Plummer referred to as the “researched life story” (page 396) in order to deliberately elicit information as to how middle-aged and older persons with SCD explained their longevity and viewed the place of the disease in their lives [21]. Our primary concern in the original study was not with how persons with SCD narratively constructed their lives, but rather with the informational content of the lives they constructed in their stories [22]. Of the 15 participants in the original study, we screened responses with a particular interest in responses to the following statement: *tell me what you think adults with sickle cell disease need to do to best take care of themselves*. Human subjects approval for this study was obtained.

### 2.1. Sampling

Participants were recruited from a list of patients being treated in an adult sickle cell program in the southeastern United States. For the original study, they were purposefully selected to represent middle-age and older adults who had lived beyond the median lifespan expected for their type of SCD. They were also selected to include both females and males as their life trajectories and SCD complications were expected to be different. We anticipated that the sample would be predominantly African American to reflect the disease profile in the US [23]. Sampling for the original study terminated at 15 participants when informational redundancy was achieved; however, only 11 individuals are included in this analysis as they were the only individuals who provided self-care recommendations. Persons with known cognitive impairment, as identified by the participants' SCD healthcare provider, were excluded from this study because they would not be able to provide the information required for participation in this study. Cognitive impairment in individuals with SCD can be related to a variety of factors including overt stroke, silent stroke localized perfusion problems, and anemia-related diffuse hypoxia [24].

### 2.2. Data Collection

The life stories of persons with SCD were elicited in moderately structured and open-ended interviews conducted in a private area of the clinic. All interviews were conducted by the first author, a nurse scientist, who is not a provider for the participants. Interviews lasted from 1 to 2 hours. 

Each participant also completed a demographic questionnaire which included general questions about age, education, income, employment, and relationship status. Participants were asked to provide specific sickle cell and other health-related information, including age of first sickle cell crisis, average number of times hospitalized with SCD crises per year, SCD complications, type of SCD, and any other medical conditions.

### 2.3. Data Analysis

All of the interviews were audio-taped and professionally transcribed verbatim. The data were subjected to thematic analysis [22]. These responses were then further analyzed for themes within each case as well as across case by sex. Two authors independently coded the transcripts initially, and then any differences were resolved by consensus.

## 3. Results

Participants included in this secondary analysis were 11 middle-aged and older adults with SCD, including 6 women and 5 men ranging in age from 48 to 72 years. Demographics of the sample are summarized in Table 1. The recommendations could be categorized by three main themes- physiological self-care, psychological self-care, and provider-related self-care. The recommendations are summarized in Table 2.

### 3.1. Physiological Self-Care Recommendations

Physiological self-care recommendations were the most prevalent self-care recommendations provided with 10 of 11 respondents providing suggestions in this area. Physiological self-care recommendations were suggestions related to taking care of the physical body and included staying warm, staying hydrated, getting enough rest, and eating “good” food. Respondents suggested that individuals with SCD should avoid smoking, drinking, and using drugs. 

Both women and men spoke about the importance of staying hydrated. A 59-year-old female with SS disease stated “Drink plenty of water. They tell me they see me, a bottle of water with me all the time. Drink, drink, drink the liquids.” Similarly, a 48-year-old male with SS disease stated that it is important “knowing what to do and what not to do.” He went on to say that one should not “overt exert yourself” and “keeping hydrated” is important.

Putting “good food” into the body was also discussed by women and men. A 58-year-old woman with SC disease stated “Well, for one thing I'd tell them you better eat right. You gotta, you gotta eat to live.” Another woman, a 53-year-old with SS disease stated “You need to do a lot of preventative measures. You need to eat right, not fast foods. You need to eat veggies and take vitamins.” A 48-year-old man with SS disease related his ability to maintain employment better than others that he knew with sickle cell to diet. He stated “Well, my work history was a little, somewhat better. I could handle myself better. I knew what to eat. I knew how far to push myself.”

### 3.2. Psychological Self-Care Recommendations

Eight of 10 respondents made recommendations in the area of psychological self-care. These recommendations focused on obtaining knowledge and understanding of the disease, listening to and learning about the body, saying prayers, and having social support in the form of someone to talk to.

The most prominent psychological self-care recommendation was to learn how to listen to one's body and learn your limits in order to learn how to adjust. A 59-year-old with SS disease stated “Your body will tell you it's tired. You listen to your body. Your body is going to tell you before you get sick. Your body is going to tell you that it's tired. And you should ··· I try to rest.” A 61-year-old who was unsure of his SCD type stated that “by paying attention to what was going on, in my life, with my body” that he has been able to take better care of himself.

Respondents also spoke about the importance of knowledge and how they often made the effort to learn more about SCD in order to take care of themselves better. During what she now describes as an avoidable hospitalization, a 53-year-old with SS disease stated that she went “on line getting research about the do's and don'ts and the ins and outs of sickle cell” because she wanted to know more. Likewise a 51-year-old male with SS disease stated “Reading. Reading up on everything about sickle cell. The more you read about it, the more you can understand your own body. The more you can do for your body. So I guess that's what it is, reading a lot of things about sickle cell.”

The final psychological self-care recommendations, praying, and talking to someone were only discussed by female respondents. A 50-year-old with SS stated “Ask God to help you. I promise you if you let him help you, you could- you don't know how long you gonna live.” Similarly, a 59-year-old with SS disease stated “Keep focused, and keep it in God's hands. Believe and trust in him. Talk to somebody.”

### 3.3. Provider-Related Self-Care

This study took place within a comprehensive sickle cell program. Respondents, both women and men, thought that an important aspect of self-care is having knowledgeable health-care providers and following their orders. A 53-year-old with SS learned over time that it is important to follow doctors' orders. She stated “I listened to the doctor, it was hard for me to adjust. I listened to the doctor, even though I didn't want to and I would still try to do some of stuff that I wanted to do, go out and party and such and I realized that I was over exerting myself. He told me that was going to happen and it happened and I was in the hospital and I had nobody to blame but myself so I really started listening to the doctor.” A 50-year-old female with SS disease stated that one should “do what the doctor say, even if you think you don't want to. Do what the doctor say.” A 48-year-old man with SS disease stated “Seeing educated people. People that are educated about sickle cell, know about sickle cell patients, and I don't have to tell them about sickle cell. And so far, I've run into doctors and nurses and I guess they've been trained. They know and I don't have to explain any of it, what sickle cell is.”

## 4. Limitations

The data are from life review interviews and therefore are influenced by participants' recall of events and feelings at the time of those events and their circumstances at the time of interview. Thus, the participants' reflection on self-care recommendations may also be influenced by his or her circumstance at the time of the interview. Additionally, these self-report interviews were conducted in one session and this is not ideal. It is also important to acknowledge that participants may have had unknown or undiagnosed cognitive impairment.

## 5. Conclusion

Although the sample size was small, it is a sufficient sample size, considering the qualitative design of the study. The researchers did not intend to make any generalizations from the findings, but to add an unexamined dimension to the explorations of the lifecourse of individuals with SCD by sharing directly the self-care recommendations of middle-aged and older adults with SCD.

Despite the noted limitations, the self-care recommendations of middle-aged and older adults living with SCD may provide guidance hope to others living with the chronic illness. A 50-year-old woman with SS disease stated it best when she said, “If you can't get no bone marrow [transplant], you got to learn to live with it.” Many middle-aged and older adults have learned to cope with the challenges of SCD over time and their life course experiences may be beneficial to others.

Although many middle-aged and older adults with SCD have lived beyond expectations, very little research has been conducted with these individuals. Thus, very little is known about their lives. However, findings are consistent with previous research that found that older African Americans are generally knowledgeable about illness and self-care and tend to have beliefs about their responsibility regarding adherence to provider recommendations [25]. Findings from this study may begin to decrease the communication gap between individuals with SCD and their providers. Additionally, it provides support for the need for a larger study wherein the limitations of this study can be minimized.

## Figures and Tables

**Table 1:** Demographic description of respondents.

Variable	Female response (*N* = 6)	Male response (*N* = 5)
Age (years)	57 (range 50–72)	54 (range 48–61)
Education	12.3 (range 5–16)	13 (range 12–15)
1st SCD crisis (age in years)	10.2* (1.5–21)	6 (range 3–9)
Average SCD crises per year (number)	2.3 (range 1–4.5)	1.2 (0–3)

Variable	*n* (%)	*n* (%)

Employment		
Disabled	4 (66.7%)	3 (60%)
Full-time	1 (16.7%)	1 (20%)
Retired	—	1 (20%)
Never employed	1 (16.7%)	—
Marital Status		
Married	2 (33.3%)	1 (20%)
Living with domestic partner	2 (33.3%)	1 (20%)
Separated	1 (16.7%)	—
Divorced	1 (16.7%)	1 (20%)
Single	—	1 (20%)
Widow	—	1 (20%)
SCD Type		
SCD-SS	4 (66.7%)	4 (80%)
SCD-SC	1 (16.7%)	—
Unsure	(14%)	1 (20%)

*Note that the average number of SCD crises was skewed by 2 respondents who reported first SCD crises at age 21.

**Table 2:** Self-care recommendation themes.

Theme	Female response frequency (*n* = 6)	Male response frequency (*n* = 5)	Total frequency (*n* = 11)
Physiological Self-care	6	4	10
Psychological Self-care	3	5	8
Provider-related Self-care	3	2	5
